# Assessment of Safety, Effects, and Muscle-Specific Accumulation of Dietary Butylated Hydroxytoluene (BHT) in *Paralichthys olivaceus*

**DOI:** 10.1155/2023/1381923

**Published:** 2023-02-02

**Authors:** Seunghan Lee, Min-Gi Kim, Sang-Woo Hur, Kumar Katya, Kang-Woong Kim, Bong-Joo Lee

**Affiliations:** ^1^Aquafeed Research Center, National Institute of Fisheries Science, Pohang 37517, Republic of Korea; ^2^Malaysian Aquaponics Research Center, Semenyih, Kuala Lumpur 43500, Malaysia; ^3^Department of Smart Fisheries Resources, Kongju National University, Yesan 32439, Republic of Korea

## Abstract

Butylated hydroxytoluene (BHT) is a commonly used antioxidant added to animal/fish feed to limit lipid autoxidation and peroxidation. Although there have been reviews and reports of BHT toxicity in animals, limited information is available with respect to the toxic effects and accumulation of BHT due to oral exposure in aquaculture species. Therefore, 120 days of feeding trial was conducted to evaluate the effects of dietary BHT on the marine fish olive flounder *Paralichthys olivaceus*. Graded levels of BHT were added to the basal diet in increments of 0, 10, 20, 40, 80, and 160 mg BHT/kg, corresponding to 0 (BHT_0_), 11 (BHT_11_), 19 (BHT_19_), 35 (BHT_35_), 85 (BHT_85_), and 121 (BHT_121_) mg BHT/kg diets, respectively. Fish with an average weight of 77.5 ± 0.3 g (mean ± SD) were fed one of the six experimental diets in triplicate groups. Growth performance, feed utilization, and survival rate were not significantly affected by the dietary BHT levels among all experimental groups, whereas BHT concentration in the muscle tissue was found to increase in a dose-dependent manner up to 60 days of the experimental period. Thereafter, BHT accumulation in muscle tissue showed a declining trend among all treatment groups. Furthermore, the whole-body proximate composition, nonspecific immune responses, and hematological parameters (except triglycerides) were not significantly influenced by the dietary levels of BHT. Blood triglyceride content was significantly higher in fish fed the BHT-free diet compared to all other treatment groups. Thus, this study demonstrates that dietary BHT (up to 121 mg/kg) is a safe and effective antioxidant without exhibiting any adverse effects on the growth performance, body composition, and immune responses in the marine fish olive flounder, *P. olivaceus*.

## 1. Introduction

Seafood is widely known as a healthy source of animal protein owing to its unique polyunsaturated fatty acid (PUFA) profile, particularly eicosapentaenoic acid (EPA, C20:5*n*-3) and docosahexaenoic acid (DHA, C22:6*n*-3) [1]. In commercial fish farming, PUFAs are supplied via feed composed of multiple ingredients, including fish oil, fishmeal, and microalgae. However, the presence of PUFAs in fish lipids makes them highly susceptible to autoxidation and peroxidative damage [2]. Such autoxidation initiates a series of other changes and produces toxic reactive oxygen species (ROS) such as superoxide, peroxides, hydroxyl radicals, and singlet oxygen in the food system. Furthermore, these products react with other native dietary nutrients and reduce the bioavailability of proteins, lipids, and vitamins. Feeding farmed species feeds containing oxidized fats may cause a reduction in growth and feed intake and sudden disease outbreaks, resulting in a higher mortality rate [3]. In seafood, such changes reportedly have detrimental effects on the nutritional profile, wholesomeness, safety, color, flavor, and texture [4], whereas consumer interest in the quality and safety of farmed fish as well as the absence of concomitant pollutants, antibiotics, and carcinogens has increased recently [5–7].

A commonly used method to improve product stability and extend the shelf-life of seafood is dietary manipulation using commercial antioxidants [8–13]. Antioxidants are highly effective in lowering or delaying the chances of oxidation of food and feed by retarding the formation of toxic oxidative products [14]. Butylated hydroxytoluene (BHT) is a commercially popular synthetic antioxidant used to preserve and stabilize the freshness, nutritive value, flavor, and color of foods and feed products [3, 15–19]. The pathway of BHT function involves blocking free radicals and converting them into stable products [17]. In order to prevent autoxidation and nutrient loss, BHT inclusion has long been practiced in animal feeds containing high levels of unsaturated fatty acids [20]. Dietary inclusion of commercial antioxidant ethoxyquin (EQ) at the rate of 150–1500 mg/kg has been reported to effectively relieve oxidative stress induced by fish oil oxidation in largemouth bass (*Micropterus salmoides*) [21]. Commercially, EQ, BHT, and butyl-hydroxyanisole (BHA) are authorized for use in animal and aquaculture feed up to a maximum concentration of 150 mg/kg. The use of BHT as a direct or indirect food/feed additive is authorized in approximately 40 countries around the world [22, 23]. However, an overdose of BHT has been reported to adversely affect various tissues and organs in rats and several other species [24–26]. Recently, Liang et al. [27] reported that exposure to BHT can influence hyperactivity and dopamine-related gene expression, which induce abnormal anxiety-associated behavior in larval zebrafish.

The marine fish olive flounder (*Paralichthys olivaceus*) is a commercially popular aquaculture species, particularly in the Republic of Korea, Japan, China, and other East Asian countries. Olive flounder aquaculture ranked first in terms of production volume and economic value (43,813 metric tons and US $369 million in 2021) in the Republic of Korea [28, 29]. Owing to its excellent taste, high market demand, and rapid growth rate, the olive flounder is also considered an ideal candidate for US land-based aquaculture [30]. The nutrient requirements of this species have been well defined, and commercial feeds are locally available and exported to other countries. Being a carnivorous species, commercial feed formulations for this species typically include high lipid levels (8%–12%) and antioxidants, such as BHT [29, 31]. With the growing concern for safety and consumer-friendly seafood, this study was undertaken to assess muscle-specific BHT accumulation, toxicity, and its effects on the growth performance, body nutritional integrity, hematological characteristics, and nonspecific immunity in *P. olivaceus*.

## 2. Materials and Methods

### 2.1. Experimental Diets

The composition and proximate analysis of the experimental diets are presented in Table 1. Six isonitrogenous and isocaloric diets were supplemented with BHT (Sigma-Aldrich, MO, USA) in increments of 0, 10, 20, 40, 80, and 160 mg BHT/kg, which corresponded to the dietary levels of 0 (BHT_0_), 11 (BHT_11_), 19 (BHT_19_), 35 (BHT_35_), 85 (BHT_85_), and 121 (BHT_121_) BHT/kg. The experimental diets were prepared by mixing the dry ingredients thoroughly in an electric mixer, followed by the addition of oil and distilled water. The mixture was passed through a screw pelleting machine (SMC-32, SL Co., Incheon, Korea) to produce pellets, which were then dried for 48 h. After drying, the pellets were broken up, sieved to the required pellet size, packed, and stored at −20°C until feeding.

### 2.2. Fish and Feeding Trial

Juvenile *P. olivaceus* were obtained from a private hatchery (Geoje-si, Gyeongsangnam-do, Republic of Korea). Juveniles (initial weight = 77.5 ± 0.3 g) were weighed and randomly stored in 300 L tanks with 35 fish per tank, with three replicate tanks per treatment. Fish were fed twice a day (09:00 and 18:00) for 120 days until apparent satiation. The feeding trial was conducted using flow-through seawater (salinity = 32.0 ± 0.4 ppt) at a flow rate of 4 L/min. Water temperature (20.7 ± 4.2°C) and dissolved oxygen levels (8.2 ± 1.3 mg/L) were maintained throughout the feeding trials. A natural photoperiod was maintained during the feeding trials, at 14 h light : 10 h dark using fluorescent lights controlled by electric timers. The fish sampling and experimental protocols were approved by the Animal Care and Use Committee of the National Institute of Fisheries Science, Republic of Korea (2021–NIFS–IACUC–7).

### 2.3. Sample Collection and Analysis

At the end of the feeding trial, all the fish were counted and weighed to calculate weight gain (WG), feed conversion ratio (FCR), and survival rate. To evaluate the pattern of BHT accumulation in the muscle between the dietary BHT levels throughout the feeding trial, six fish (starved for 24 h before sampling) from each treatment (two fish from each of three replicate tanks per treatment) were collected at the starting point and at the end of the 60 days and 120 days of feeding trials. The collected fish were euthanized with tricaine methanesulfonate (MS-222, 200 mg/L, buffered to pH 7.4) and dissected to sample the dorsal muscle. Muscle samples were snap-frozen in liquid nitrogen and stored at −80°C until BHT analysis. At the end of the feeding trials, blood was collected from the caudal vein of six randomly selected fish per aquarium using a 5 mL heparinized syringe. The collected blood samples were centrifuged at 1500 × *g* for 15 min at 4°C, and plasma was collected for investigating blood chemistry and immune responses. Additionally, four fish per tank were euthanized and stored at −20°C for whole-body proximate composition analyses after the feeding trials.

BHT determination was performed on muscle samples according to the AOAC official method [32], which measures BHT after sample dissolution in hexane and extraction of antioxidants into acetonitrile. The concentrated acetonitrile solution was diluted with an equal volume of isopropanol. The diluted solution was injected into a high-performance liquid chromatography (HPLC) machine, with detection at 280 nm using a UV detector (Agilent 1260 System, Agilent Technologies, Waldbronn, Germany).

Superoxide dismutase (SOD), glutathione peroxidase (GPx), immunoglobulin M (IgM), and lysozyme (LZM) activity was determined using ELISA kits according to the manufacturer's protocols (SOD, cat. No. MBS705758; GPx, cat. No. MBS705700; IgM, cat. No. MBS700823; LZM, cat. No. MBS705758, MyBioSource, USA). Plasma aspartate transaminase (AST), glucose, total protein, total cholesterol, and triglyceride levels were determined using an automated blood analyzer (Clinical Chemistry Analyzer; Thermo Fisher Scientific, Finland), following the manufacturer's protocol.

The crude protein, crude lipid, ash, and moisture content of the experimental diets and whole body were analyzed according to standard methods [32]. Briefly, moisture content was determined by oven drying at 105°C, crude protein content was analyzed using the Kjeldahl method, crude lipid content was analyzed by Soxhlet extraction using a Soxhlet 1046 system (Tacator AB, Sweden), and ash content was determined by incineration (550°C) for 4 h.

### 2.4. Statistical Analysis

The homogeneity of variance was tested using Levene's test. All data were subjected to one-way analysis of variance (ANOVA) using SAS version 9.3 (SAS Institute, Cary, NC, USA). When a significant effect was observed, post hoc Duncan's test was performed to compare the treatment means.

## 3. Results

### 3.1. Growth Performance

Table 2 summarizes the growth performance (final body weight, weight gain %, FCR, and survival rate) of olive flounders fed different levels of BHT-supplemented diets for 120 days. Butylated hydroxytoluene had no significant effects on the growth and survival rate of *P. olivaceus* (*P* > 0.05). Experimental diets were well accepted and consumed by the experimental fish, as evidenced by the excellent FCR (1.01–1.04) and survival rate (97%–100%) recorded among different dietary treatments. Average weight gain ranged from 523% to 550% among different treatment groups, without any significant differences (*P* < 0.05), whereas BHT intake per fish significantly increased in a dose-dependent manner and was significantly higher in the group fed the BHT_121_ diet, followed by the BHT_85_, BHT_35_, BHT_19_, BHT_11_, and BHT_0_ diets (*P* < 0.05).

### 3.2. Muscle-Specific BHT Accumulation in *P. olivaceus*

**Figure 1:**
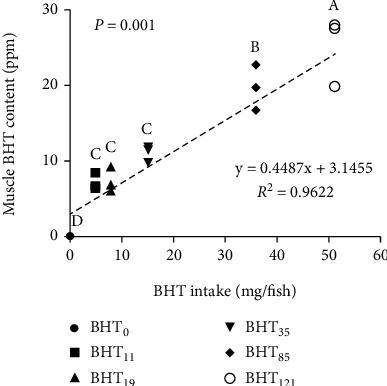
Muscle-specific BHT content according to BHT intake over a 120-day growth trial in olive flounder (*Paralichthys olivaceus*). Values are means from triplicate groups of fish, and the values in each row with different superscripts are significantly different (*P* < 0.05).

**Figure 2:**
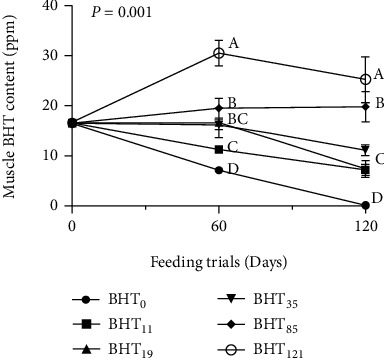
Muscle-specific BHT accumulation in olive flounder (*Paralichthys olivaceus*) fed experimental diets over a 120-day growth trial. Values are means from triplicate groups of fish where the values in each row with different superscripts are significantly different (*P* < 0.05).

Figures 1 and 2 show BHT accumulation in the muscle tissues of olive flounder fed various levels of BHT-supplemented diets. The muscle BHT concentrations increased with a corresponding increase in the BHT intake up to 60 days (*P* < 0.05). Thus, the highest BHT accumulation was recorded in the fish fed the BHT_121_ diet followed by those fed the BHT_85_, BHT_35_, BHT_19_, BHT_11_, and BHT_0_ diets.

In contrast, the effects of culture duration on muscle-specific BHT accumulation were clearly observed (Figure 2). At the end of the 60-day culture period, the BHT concentration among the fish fed BHT_121_ diet was significantly higher than those of fish fed BHT_85_ followed by BHT_35_, BHT_19_, BHT_11_, and BHT_0_ diets (*P* < 0.05), respectively. However, no significant differences were detected in the muscle tissue BHT content among the groups of fish fed the BHT_35_, BHT_19_, and BHT_11_ diets (*P* > 0.05). Interestingly, after 60 days, the BHT concentrations among all treatment groups tend to gradually decline; however, at the end of 120 days, the muscle BHT content of the flounders fed the BHT_121_ diet was significantly higher than those of fish fed other experimental diets (*P* < 0.05).

### 3.3. Nonspecific Immune Responses

Table 3 shows the nonspecific immune responses of the experimental fish fed diets supplemented with different levels of BHT for 120 days. The immune parameters SOD, GPx, IgM, and lysozyme activity responded differently to the dietary treatments. Although numerical differences were observed between the treatments, BHT had no significant effects on the overall nonspecific immune responses in *P. olivaceus* (*P* > 0.05).

### 3.4. Hematological Parameters

Table 4 shows the hematological parameters of the fish that were fed diets supplemented with various levels of BHT for 120 days. It appeared that BHT had no significant effects on the hematological parameters (*P* > 0.05) except for decreased serum triglycerides in *P. olivaceus*. The triglyceride content of the fish fed the BHT_11_, BHT_19_, BHT_85_, and BHT_121_ diets was significantly lower than that of fish fed the BHT_0_ diet (*P* < 0.05). However, the triglyceride content of the BHT_35_ groups was not significantly different from that of the other experimental groups (*P* > 0.05). Although numerical differences were recorded in the AST, glucose, total protein, and cholesterol content among the different treatment groups, no clear trend could be observed (*P* > 0.05).

### 3.5. Whole-Body Proximate Composition

The effects of different levels of BHT-supplemented diets on the whole-body proximate composition of the olive founders are shown in Table 5. Butylated hydroxytoluene had no significant effects on the whole-body proximate composition, including moisture, crude protein, crude lipid, and crude ash among the treatment groups (*P* > 0.05).

## 4. Discussion

The growing safety concerns associated with seafood production and consumption require a clear understanding of both nutrients and contaminants throughout the production chain. In recent years, the production of consumer-friendly seafood using feeds that are low in contaminants and antioxidants has received considerable attention. The synthetic antioxidant BHT, through the carry-over process from fish feed, can have toxic and negative health impacts on both fish and consumers [33]. However, observations from the current 120-day-long study showed no beneficial or adverse effects of BHT inclusion on olive flounder fed up to a concentration of 121 mg/kg.

As evidenced by the excellent survival rate (97%–100%), growth performance, and FCR, the experimental diets were well accepted and consumed by the fish in all treatment groups (Table 4). Studies on the effects of dietary BHT in commercial aquaculture species are scarce but have grown in the last few years. In a two-decade-old study, Bautista-Teruel and Subosa [3] examined the effects of BHT inclusion on the diet of marine shrimp, *Penaeus monodon*, reared at various temperatures for 60 days. These authors observed that dietary BHT resulted in a substantial improvement in the growth of juvenile shrimp only after 45 days, although the number of treatments was limited in this study. In contrast, in a more recent study, Yu et al. [34] reported no significant improvement in the growth of largemouth bass (*Micropterus salmoides*) fed various levels of BHT-supplemented diets for 10 weeks. Similarly, in the present study, no evidence was found suggesting any positive/negative impact of BHT inclusion up to 121 mg/kg (BHT_121_) on the growth performance of *P. olivaceus* reared for 120 days.

Interestingly, published reports also suggest weight loss in rats fed BHT-supplemented diets at 125–150 mg/kg body weight [24]. Similarly, growth reduction in rats fed BHT-supplemented diets at 1,500 mg/kg body weight has also been reported [24]. Notably, carnivorous fish, such as olive flounder and largemouth bass, have comparatively higher lipid requirements than rats and most mammals [35, 36] and may also have a higher tolerance to dietary antioxidants such as BHT. The results of the present study are in agreement with a previous study [34], suggesting no beneficial or adverse impacts of dietary BHT on the growth performance of *P. olivaceus*.

The residue levels of synthetic antioxidants in food products, including seafood, have become a matter of serious concern. Despite the urgency driven by the growing awareness and interest in healthy human food, only limited published data are available on the residue levels of synthetic antioxidants in commercial fish fillets [21]. A previous study [37] found BHT to be the most abundant antioxidant in Atlantic salmon fillets (420 mg/kg) followed by ethoxyquin dimer (EQDM) and BHA. Similarly, BHT was found to be the most prominent antioxidant in Atlantic salmon, Atlantic halibut, and rainbow trout fillets [21]. In the present study, an increase in whole-body BHT concentrations in fish was observed with the corresponding increase in dietary inclusion (Figure 1); however, whole-body BHT accumulation gradually declined among all the treatment groups after 60 days of culture (Figure 2). Notably, the measured BHT concentrations in olive flounder were lower than those reported for Atlantic salmon [37]. This may reflect differential BHT accumulation in the fish body due to differences in the fish size, species, and dietary inclusion level.

Interestingly, BHT accumulation was also observed in the BHT-free (BHT_0_) diet treatment. Although 1,000–4,000 mg/kg of BHT is typically included in fishmeal production to prevent spontaneous combustion during overseas transport and storage [21, 38], the fishmeal used in this experiment was BHT-free (Table 1). Therefore, the accumulation of BHT in the tissues of the BHT_0_ treatment group likely reflects accumulation during the 10-day acclimation period, when all the fish were fed the BHT_35_ diet. Overall, BHT accumulated in the muscle tissues of *P. olivaceus* in a dose-dependent manner during the first 60 days of the culture period and afterwards gradually declined.

In contrast to BHT accumulation in muscle issues, there was no difference in whole-body moisture, crude protein, crude lipid, and ash contents among the different treatment groups (Table 5). Among the few reports on dietary BHT in aquaculture species, only one study has reported the effects of BHT on the proximate composition and muscle tissues of fish [23]. Importantly, further long-term studies are needed to better understand the effects of BHT dose and culture period on the pattern of BHT accumulation in fish tissues and the whole-body nutrient profile.

Nonspecific immunity is the major defense system in fish and shellfish and plays a vital role in acquiring immune responses and homeostasis through a system of receptor proteins. In aquaculture, optimized antioxidants and immunity are important for combating stress, maintaining health, and ensuring optimal survival and growth [39, 40]. Among them, SOD is one of the most important antioxidant enzymes, known for its efficiency in controlling reactive oxygen species in cells and catalyzing the dismutation of hydrogen peroxide and normal oxygen from superoxide radicals [41, 42]. Lysozyme is another cationic protein synthesized in the liver and extrahepatic sites in aquaculture species and is well known for its bacteriolysis, opsonization, and antimicrobial activity [43], whereas IgM is the only class of serum immunoglobulin reported in fish and is known to play a key role in a variety of immune processes, including the neutralization of pathogenic bacteria, viruses, and toxins [44, 45]. Substantial changes in these parameters could be a potential indicator of stress in fish caused by biotic or abiotic factors. In the present study, nonspecific immune parameters (Table 3) were recorded in the optimal range and were not significantly affected by the dietary BHT inclusion up to 121 mg/kg (BHT_121_). Similarly, Yu et al. [34] reported no significant variation in the levels of SOD and other antioxidants in largemouth bass fed a BHT-supplemented diet of up to 1,500 mg/kg.

Hematological parameters, such as triglycerides, total protein, AST, cholesterol, and glucose, are known to be promising indicators of animal health [42, 46, 47]. In the present study, the AST, total protein, glucose, and cholesterol contents of *P. olivaceus* were not significantly affected by the dietary BHT treatments. In comparison, triglyceride content tended to decrease as the BHT concentration increased and was lowest among the BHT_121_ treatment group. Decreasing plasma triglyceride levels with a corresponding increase in BHT levels could be a potential indicator of enhanced lipid metabolism [34]. Indeed, similar observations in the hematological parameters of largemouth bass have been reported [34]. It is worth mentioning that a previous study with rats reported a decrease in plasma triglyceride levels caused by dietary BHT inclusion [48]. Likewise, another study with BHA also reported a decrease in triglyceride levels with a corresponding increase in dietary BHA inclusion [49]. A possible reason for such observations with triglyceride levels has been attributed due to the antioxidant activity of BHT, which may alleviate the abnormal intracellular events involved in lipid metabolism [30, 32, 33]. Furthermore, considerable changes in the hematological parameters of aquatic species have been reported following exposure to different stress conditions caused by pollutants, diseases, and hypoxia [50]. Hence, any stressful condition caused by poor nutrition could have a visible impact on the hematological characteristics of fish [51]. However, in the present study, hematological parameters, including AST, total protein, glucose, and cholesterol content, were not considerably affected by BHT supplementation up to 121 mg/kg.

## 5. Conclusions

The inclusion of BHT in the diet of *P. olivaceus* up to 121 mg/kg was found to be safe and effective with no beneficial/adverse effects on the fish growth, nonspecific immunity, and whole-body nutrient profiles. However, observations for the hematological characteristics showed an adverse effect on the triglyceride levels with a corresponding increase in the dietary BHT levels. Nevertheless, the observation of BHT accumulation in the fish muscle tissues during the first 60 days of exposure and its subsequent gradual decline warrants further examination using long-term farm trials for *P. olivaceus* and other important aquaculture species.

## Figures and Tables

**Table 1:** Ingredient composition of the experimental diets (g kg^−1^ of as-is basis).

Ingredients	Diet no. (analyzed BHT level, mg kg^−1^ diet)^1^					
	BHT_0_	BHT_11_	BHT_19_	BHT_35_	BHT_85_	BHT_121_
BHT-free fishmeal^2^	70.00	70.00	70.00	70.00	70.00	70.00
Soybean meal	10.00	10.00	10.00	10.00	10.00	10.00
Tapioca starch	8.000	7.999	7.998	7.996	7.992	7.984
Wheat flour	3.30	3.30	3.30	3.30	3.30	3.30
Fish oil^2^	4.00	4.00	4.00	4.00	4.00	4.00
Lecithin	0.50	0.50	0.50	0.50	0.50	0.50
Betaine	0.50	0.50	0.50	0.50	0.50	0.50
Taurine	0.50	0.50	0.50	0.50	0.50	0.50
Mineral mixture^3^	1.50	1.50	1.50	1.50	1.50	1.50
Vitamin mixture^4^	1.20	1.20	1.20	1.20	1.20	1.20
Choline	0.50	0.50	0.50	0.50	0.50	0.50
BHT^5^	0	0.001	0.002	0.004	0.008	0.016
Total	100.0	100.0	100.0	100.0	100.0	100.0
*Analyzed nutrient compositions (as-is basis)*						
Moisture (%)	2.55	2.50	2.36	2.89	2.30	2.13
Crude protein (%)	48.30	48.59	48.69	47.73	48.18	48.35
Crude lipid (%)	12.05	12.24	11.96	12.02	11.70	11.72
Crude ash (%)	17.33	17.12	16.96	17.54	17.63	17.47
Gross energy (cal/g)	4584	4554	4599	4500	4542	4520
BHT (ppm)	0.00	11.33	19.19	35.14	84.96	121.01

^1^BHT was added in increments of 0, 10, 20, 40, 80, and 160 mg BHT/kg diet (analyzed levels: BHT_0_, BHT_11_, BHT_19_, BHT_35_, BHT_85_, and BHT_121_, respectively). ^2^Suhyup Feed Co., Uiryeong, Republic of Korea. ^3^The mineral premix contained the following amounts diluted in cellulose (g/kg premix): NaCl, 30.3; MgSO_4_-7H_2_O, 95.6; NaH_2_PO_4_-2H_2_O, 60.8; KH_2_PO_4_, 167.3; CaH_4_(PO_4_)_2_-H_2_O, 94.7; ferric citrate, 20.7; ZnSO_4_-7H_2_O, 15.3; Ca-lactate, 212.8; CuCl, 0.14; AlCl_3_-6H_2_O, 0.105; KI, 0.105; Na_2_Se_2_O_3_, 0.01; MnSO_4_-H_2_O, 1.4; and CoCl_2_-6H_2_O, 0.7. ^4^The vitamin premix contained the following amounts diluted in cellulose (g/kg premix): L-ascorbic acid, 171.1; myo-inositol, 181.8; DL-a-tocopheryl acetate, 18.9; niacin, 36.4; p-aminobenzoic acid, 18.2; Ca-D-pantothenate, 12.7; riboflavin, 9.1; thiamin hydrochloride, 2.7; pyridoxine hydrochloride, 1.8; menadione, 1.8; retinyl acetate, 0.73; folic acid, 0.68; D-biotin, 0.27; and cholecalciferol, 0.003. ^5^Butylated hydroxytoluene (CAS No. 128-37-0, Product No. PHR1117), Sigma-Aldrich, St. Louis MO, USA.

**Table 2:** Growth performance of olive flounder (*Paralichthys olivaceus*) fed experimental diets for 120 days^1^.

	BHT_0_	BHT_11_	BHT_19_	BHT_35_	BHT_85_	BHT_121_	*P* value
IBW^2^	77.4 ± 0.2	77.5 ± 0.4	77.4 ± 0.4	77.4 ± 0.1	77.4 ± 0.4	77.4 ± 0.4	0.943
FBW^3^	502.9 ± 3.0	496.9 ± 16.8	490.7 ± 16.0	494.6 ± 22.0	483.5 ± 13.5	482.3 ± 5.0	0.492
WG (%)^4^	550.0 ± 2.3	540.8 ± 18.8	532.4 ± 19.3	538.8 ± 27.5	524.4 ± 19.8	523.2 ± 7.3	0.460
FI (g/fish)^5^	431.9 ± 18.2	429.7 ± 12.3	424.7 ± 5.9	430.7 ± 15.5	423.3 ± 9.5	423.1 ± 11.2	0.922
BHT intake (mg/fish)	0.0^f^	4.87 ± 0.14^e^	7.86 ± 0.51^d^	15.1 ± 0.5^c^	36.0 ± 0.8^b^	51.2 ± 1.4^a^	<0.001
FCR^6^	1.01 ± 0.04	1.03 ± 0.02	1.03 ± 0.03	1.03 ± 0.02	1.04 ± 0.01	1.04 ± 0.02	0.864
Survival (%)^7^	100	98.1 ± 1.6	98.6 ± 2.0	97.1 ± 4.9	98.1 ± 1.6	99.0 ± 1.6	0.943

^1^Values are means from triplicate groups of fish where the values in each row with different superscripts are significantly different (*P* < 0.05). ^2^Initial body weight (IBW g/fish). ^3^Final body weight (FBW g/fish). ^4^Weight gain (WG%) = (FBW − IBW) × 100/IBW. ^5^Feed intake (FI, g/fish). ^6^Feed conversion ratio (FCR) = dry feed intake/wet weight gain. ^7^Survival rate (%) = (total fish–dead fish) × 100/total fish.

**Table 3:** Nonspecific immune responses of olive flounder (*Paralichthys olivaceus*) fed experimental diets for 120 days^1^.

	BHT_0_	BHT_11_	BHT_19_	BHT_35_	BHT_85_	BHT_121_	*P* value
SOD (ng/mL)	1.73 ± 0.07	1.58 ± 0.32	1.58 ± 0.21	1.56 ± 0.35	1.67 ± 0.24	1.46 ± 0.20	0.824
GPx (*μ*U/mL)	749 ± 48	703 ± 27	682 ± 61	698 ± 70	694 ± 68	681 ± 32	0.663
IgM (*μ*g/mL)	5.55 ± 0.98	5.70 ± 0.57	5.18 ± 0.98	5.67 ± 0.38	4.78 ± 0.31	5.57 ± 0.95	0.641
Lysozyme (unit/mL enzyme)	19.9 ± 7.9	24.9 ± 0.5	19.6 ± 7.9	20.8 ± 3.5	15.9 ± 8.1	12.3 ± 7.6	0.335

^1^Values are means from triplicate groups of fish where the values in each row with different superscripts are significantly different (*P* < 0.05).

**Table 4:** Hematological parameters of olive flounder (*Paralichthys olivaceus*) fed various levels of BHT-supplemented diets for 120 days^1^.

	BHT_0_	BHT_11_	BHT_19_	BHT_35_	BHT_85_	BHT_121_	*P* value
AST (U/L)	40.1 ± 9.9	32.1 ± 5.0	33.4 ± 2.3	34.8 ± 5.1	29.7 ± 8.2	31.2 ± 7.7	0.608
Glucose (mg/dL)	33.3 ± 8.6	37.3 ± 14.3	38.2 ± 15.1	34.3 ± 10.0	46.2 ± 26.2	26.6 ± 14.0	0.792
Total protein (mg/dL)	4.35 ± 0.21	4.18 ± 0.15	4.06 ± 0.21	4.25 ± 0.38	4.31 ± 0.12	4.17 ± 0.15	0.641
Cholesterol (mg/dL)	377 ± 19	346 ± 31	381 ± 5	386 ± 13	352 ± 99	362 ± 26	0.834
Triglyceride (mg/dL)	515 ± 99^a^	229 ± 49^b^	335 ± 90^b^	376 ± 123^ab^	296 ± 73^b^	224 ± 99^b^	0.020

^1^Values are means from triplicate groups of fish where the values in each row with different superscripts are significantly different (*P* < 0.05).

**Table 5:** Whole-body proximate composition (%, as-is basis) of olive flounder (*Paralichthys olivaceus*) fed experimental diets for 120 days^1^.

	BHT_0_	BHT_11_	BHT_19_	BHT_35_	BHT_85_	BHT_121_	*P* value
Moisture	71.9 ± 0.4	71.6 ± 0.7	71.6 ± 1.0	71.2 ± 0.3	70.9 ± 0.6	71.1 ± 0.8	0.652
Crude protein	18.7 ± 0.2	18.5 ± 0.1	18.5 ± 0.2	18.7 ± 0.1	18.5 ± 0.3	18.7 ± 0.1	0.515
Crude lipid	6.09 ± 0.54	6.29 ± 0.62	5.83 ± 0.65	6.53 ± 0.23	6.41 ± 0.33	6.50 ± 0.52	0.723
Crude ash	3.45 ± 0.10	3.47 ± 0.49	3.41 ± 0.07	3.36 ± 0.26	3.18 ± 0.51	3.24 ± 0.61	0.964

^1^Values are means from triplicate groups of fish where the values in each row with different superscripts are significantly different (*P* < 0.05).

## Data Availability

The data used to support the findings of this study are included in the article.
